# EpCAM expression negatively regulates E-cadherin function in colorectal carcinomas

**DOI:** 10.3332/ecancer.2023.1569

**Published:** 2023-07-06

**Authors:** Uchenna Simon Ezenkwa, Gabriel Olabiyi Ogun, Mbwas Isaac Mashor, Olufemi John Ogunbiyi

**Affiliations:** 1 Federal Medical Centre Azare, Azare 751101, Bauchi, Nigeria; 2 Department of Pathology, University College Hospital, Ibadan 200285, Oyo, Nigeria; 3 Department of Pathology, Bringham University, Jos 930105, Plateau, Nigeria; a https://orcid.org/0000-0002-7022-8268; b https://orcid.org/0000-0002-8748-2879

**Keywords:** epithelial cell adhesion molecule, E-cadherin, colorectal carcinoma

## Abstract

**Background:**

This study aimed to characterise epithelial cell adhesion molecule (EpCAM) expression patterns in colorectal carcinomas (CRC) from Nigerian patients, its association with E-cadherin and tumour characteristics, to forecast patient selection for anti-EpCAM therapy among whom no data existed previously.

**Methods:**

Tissue microarray blocks of formalin-fixed and paraffin-embedded CRC tissues, with their non-cancer margins of resection, were sectioned and stained with EpCAM and E-cadherin primary antibodies. Scoring for antibody staining was done semiquantitatively by combining staining proportion and intensity. The outcome was correlated with patient age, gender and tumour histological parameters with *p* ≤ 0.05 regarded as statistically significant.

**Results:**

Sixty-three carcinoma tissues had staining status for the two markers and were included in this study. Of these, 36 (57.1%) showed positive EpCAM expression (immunoscore ≥3) out of which 83% (30/36 positive cases) were overexpressed (combined immunoscore ≥4) while 12 (19%) tissues were positive for E-cadherin. Non-tumour margins of resection tissues showed less EpCAM positivity in 24% (6/25) of histospots. The difference in staining between tumour and non-tumour margin tissues with EpCAM was significant (*p* < 0.001). Also, EpCAM overexpression was significantly associated with reduced E-cadherin (*p* < 0.035) expression in tumour cells. Tumour extent within the gut wall was equal (50% each) for early and late pT stages among EpCAM overexpressing tumours but two-thirds (8/12) of cases expressing E-cadherin had later pT stage paradoxically, while distant metastasis was negligible among tumours bearing both markers. Also, tumours overexpressing EpCAM had significant association with tumour-associated lymphocytes (*p* < 0.02 each).

**Conclusion:**

CRC in this study preferentially overexpress EpCAM over E-cadherin whose strong cell-cell contact inhibitory role is weakened even when expressed, resulting in further local tumour spread. This, and the observed immune response, supports targeted therapy among eligible patients.

## Introduction

Colorectal carcinoma (CRC) is the third most common cancer globally after female breast cancer and lung cancer [1]. Whereas its incidence is stabilising or declining in Western nations, Nigeria and other African countries have continued to witness a steady rise in its incidence with reports from Nigeria suggesting a three-fold rise in four decades [1–3]. The disease demographic shows disparity in age of occurrence and late stage at presentation [2, 4, 5]. Besides delay in seeking care [6], molecular characteristics of the tumour cells such as changes in the activities or expression of cellular adhesion molecules may result in enhanced tumour aggressiveness [7]. First discovered as a tumour antigen in CRC, epithelial cell adhesion molecule (EpCAM) has been shown to down-regulate E-cadherin expression and/or its function in cancer cells, thereby promoting tumour spread [8, 9]. Additionally, E-cadherin, by its function, regulates Wnt/β-catenin signalling thereby regulating cell turnover. Deregulated E-cadherin would therefore promote tumour cell proliferation in addition to metastasis [10]. These properties would project EpCAM as a poor prognostic tumour marker, but its role as a weak cell aggregator among fibroblasts would suggest a favourable prognosis under differing conditions [11]. EpCAM is a druggable target with many on-going clinical trials and thus, patients with tumours expressing this marker are likely to benefit from this therapy in the future [11]. However, to our knowledge, no preliminary data on EpCAM expression by CRC has been documented among our patient cohort. Investigating this biomarker in our patients with CRC would help to establish baseline data on its expression pattern and association with histopathological prognostic variables thereby filling the knowledge gap from our population and, forecasting patient selection for anti-EpCAM therapy in the future.

## Material and methods

This was a retrospective descriptive study of CRC resections diagnosed over a 10-year period (January 2008–December 2017) conducted at a University Hospital in Southwestern Nigeria. All patients that had suitable formalin fixed paraffin-embedded (FFPE) cancer tissues at the time of the study were included in the study. Cases with missing FFPE tissue blocks, or unrepresentative/inadequate tissues within the paraffin wax, were excluded. Haematoxylin and eosin-stained slides of the tumour tissues were reviewed to document tumour histology, grade, stage, tumour-associated lymphocytes (TAL) status, lymphovascular invasion and perineural invasion [12, 13] in addition to selecting appropriate FFPE tissue blocks for the preparation of tissue microarray (TMA) recipient blocks. In addition, non-tumour-bearing margins of resection tissues from the colectomies were also reviewed for comparison. The clinicopathological data age, sex and site of tumour contained in the pathology request forms were retrieved using a proforma. Institutional Review Board approval was obtained prior to the commencement of this study (see below) and all procedures were conducted according to the Helsinki declaration on studies involving human subjects of 1964 and its later amendments.

### TMA preparation

The Kononen TMA technique was used in the construction of the TMA blocks in this study [14]. H&E stained slides retrieved above were viewed using the Fisherbrand™ 425 series zoom stereoscopic microscope and a slide marker was used to map out representative areas of tumour on the slides. The marked slides were then used to map out the representative tumour areas on the FFPE CRC tumour tissue blocks. Recipient paraffin TMA blocks (PTMA) were made and wells (diameter of 1 mm) were constructed in each of them using a quick-ray manual tissue microarrayer. Paraffin tissue core biopsy specimens (diameter of 1 mm) were then obtained from the representative areas on the marked FFPE CRC tumour tissue blocks and also from the non-tumour FFPE tissues (donor) blocks and arranged to fill up the wells in the recipient PTMA blocks using the quick ray manual tissue microarrayer. A table map was drawn to represent the precise location of each tumour. There was a total of 109 FFPE tissue blocks, each derived from an individual patient and corresponding to 109 histospots produced on the 2 TMA recipient paraffin blocks. Microtome sections were cut at 4 μm thickness, stained with H&E stain and evaluated for adequacy of tumour tissue.

### Tissue slide preparation, antigen retrieval and immunohistochemical staining

Sections (4 μm thick) from the TMA tissue blocks were cut and mounted onto electrostatically-charged slides. Deparaffinisation was by xylene treatment. Antigen retrieval was done using the heat treatment method with incubation in 0.01% buffered Ethylenediaminetetraacetic acid (EDTA) for 5 minutes at 95°C. To block the activities of endogenous peroxidase, the tissue slides were incubated for five minutes in 1% hydrogen peroxidase and subsequently washed in phosphate-buffered saline.

The indirect immunoperoxidase staining technique was adopted for the immunohistochemical staining of the tissues. Monoclonal primary antibodies were obtained from Santa Cruz Biotechnology, Inc. Bergheimer Str.89-2, 69115 Heidelberg Germany with catalogue numbers as follows: Ep-CAM, sc-25308; and E-cadherin, sc-56527. Colour development was visualised by incubating the specimen in a hydrogen peroxide-diaminobenzidine mixture mixed in 0.1 M Tris-HCL PH 7.6.

#### EpCAM IHC staining interpretation

Antigen expression was defined as specific when a staining signal was present on the tumour cell membrane. Similar to the Allred score in the evaluation of oestrogen receptor positivity, EpCAM expression was evaluated by calculating a total immunostaining score (TIS) as the product of a proportion score (PS) and an intensity score (IS). The PS describes the estimated fraction of positively stained tumour cells (0, none; 1, <10%; 2, 10%–50%; 3, 51%–80%; 4, >80%) [15]. The IS represents the estimated staining intensity as compared with control cell lines (0, no staining; 1, weak; 2, moderate; 3, strong). The TIS (TIS = PS × IS) ranges from 0 to 12 with only nine possible values (that is, 0, 1, 2, 3, 4, 6, 8, 9 and 12). EpCAM IS score of 0–2 was regarded as absent or weak expression, scores 3, 4 and 6 were moderate expression while 8, 9 and 12 was strong expression. Absent or weak expression was defined as negative, and moderate or strong expression was defined as positive expression. Furthermore, TIS > 4 was taken as overexpression.

#### E-cadherin IHC staining interpretation

Immunostaining intensity and proportion of cellular staining was determined semiquantitatively: the intensity score represents the average staining intensity (0 = negative, 1 = weak, 2 = intermediate and 3 = strong) [16]. The proportion of cellular staining was scored 3 when 60% or more of the cancer cells showed staining; 30%–59% of tumour cells staining positive was scored as 2; score 1 was staining of 5%–29% of the cells. Positivity in less than 5% of cells qualifies for score 0. The protein expression was regarded as positive regardless of whether the expression is mainly at the apical portion of the cell or evenly distributed around the cell membrane [17]. The overall score was the addition of the two scores. An aggregate score of 0–2 was defined as a negative expression while score 3–6 was regarded as positive.

### Histospots evaluation

Seventy-three (67%) of the 109 histospots had representative tumour tissues fit for evaluation of EpCAM antibody expression, and 65 (60%) histospots for E-cadherin. The others were unevaluable due to the insufficient number of epithelial tumour cells, extensive necrosis, poor tumour preservation or loss of tissue on the TMA. Likewise, the normal (margin of resection) colonic tissue recipient paraffin blocks yielded evaluable 25 (61%) out of 41 histospots created.

Thereafter, we identified 63 histospots that when sectioned and stained immunohistochemically on different slides, had adequate tumour cells that could each be evaluated for the presence or lack of EpCAM and E-cadherin expression simultaneously and these were included in the final analysis.

### Statistical analysis

Derived data were analysed statistically using Statistical Package for the Social Sciences version 20. Descriptive statistics was used to determine the proportion of each categorical variable, mean and median of continuous variables. Chi-square test was used to determine association between categorical variables. Differences in means of age were determined using student *t*-test statistics. The level of statistical significance was set at ≤0.05.

## Results

**Figure 1.**
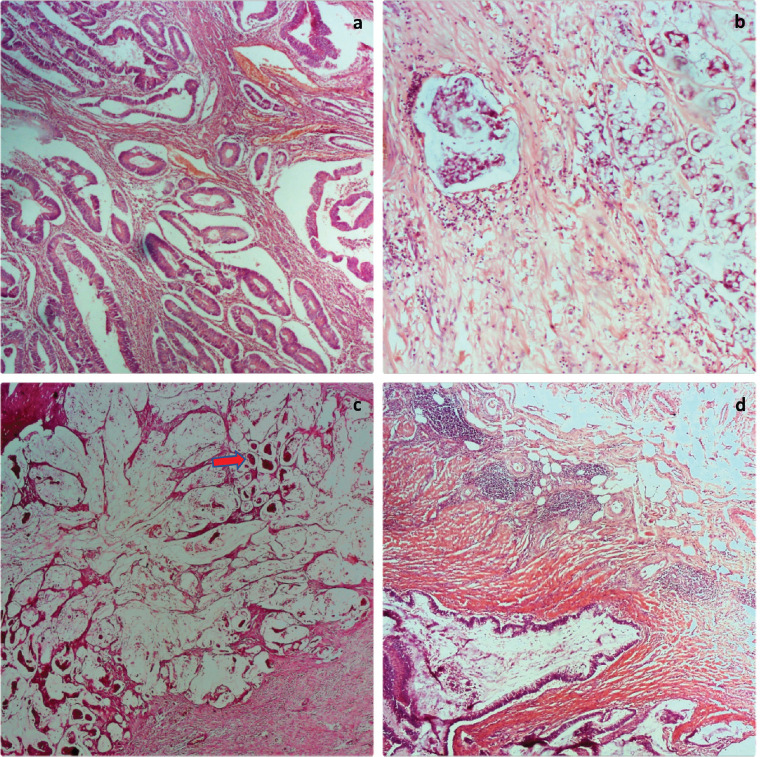
Photomicrographs of tumour grades. (a): Well differentiated adenocarcinoma; (b): Signet ring carcinoma; (c): mucinous adenocarcinoma with clusters of tumour cells floating in pools of mucin (red arrow); and (d): mucinous adenocarcinoma with Crohn-like lymphocytic aggregates at the tumour invasive border. H&E ×100.

Of the 63 cases, there were 33 females and 30 males giving a female-male ratio of 1.1:1. The mean age of the patients was 56.6 ± 18 years. Table 1 shows other clinicopathological parameters of the patients and the tumours. Tumour location was available for 55 tumours, 63.3% (35/55) of these were in the left colon and rectum. Histological subtype was mostly adenocarcinoma not otherwise specified (NOS), predominantly well differentiated. No case of undifferentiated adenocarcinoma was seen. pT and M staging were complete for all the cases, 10 (15.9%) tumours had no nodal status and were accordingly classified as Nx. Overall, 61.9% of the tumours were of early stage based on TNM staging. Micrographs showing well-differentiated adenocarcinoma (NOS), signet ring cell carcinoma and mucinous carcinoma are shown in Figure 1a–c while Figure 1d shows Crohn-like inflammation.

### EpCAM and E-cadherin immunostaining

**Figure 2.**
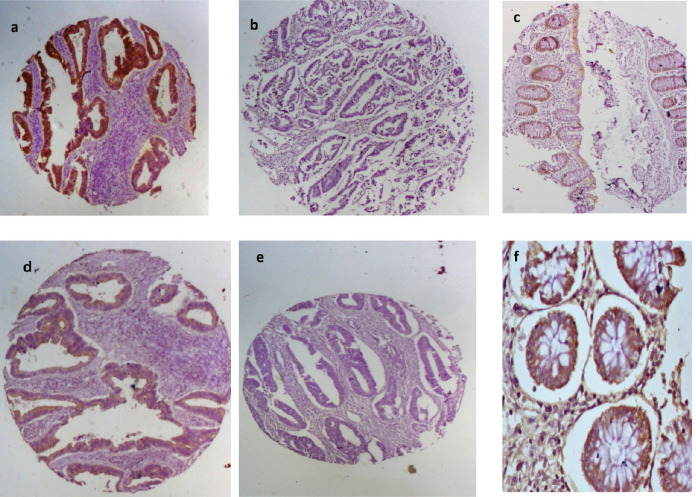
EpCAM and E-cadherin immunohistochemical staining patterns for the biomarkers. First row shows (a): positive and (b): negative EpCAM expression in CRC and (c): moderate staining in normal colon mucosa. Second row shows E-cadherin (d): positivity and (e): negativity in CRC tissues and (f): strong positivity in normal colon mucosa.

Positive EpCAM expression (total immunoscore ≥3) was observed in 36 (57.1%) of the cases. Normal colon tissue showed positive EpCAM expression in 6 (24%) of the 25 histospot specimens. Among the EpCAM-positive tumours, 83.3% (30/36 positive cases) showed marker overexpression (total immunoscore ≥4). Twenty of the negative cases had no immunostaining while the remaining seven had total immunoscore of 2 (five cases) and score of 1 (two cases). E-cadherin expression occurred in greater proportion among non-tumour-bearing colon tissues being present in 15 (60%) out of 25 tissues from the margins of resection. Tumour tissues had positive expression in 12 (19%) of tumours. Half of the positive tumours showed moderate expression (aggregate immunoscore 3–4) whilst the other half had strong E-cadherin expression (aggregate immunoscore 5–6). There was a significant association between EpCAM and E-cadherin expression in these tumour cells (*p* =0.035)*.* The positive and negative EpCAM and E-cadherin immunostains are shown in Figure 2.

### Association between EpCAM and E-cadherin and clinicopathological variables

Chi-square test statistic results of the association between the immunomarkers and clinicopathological parameters are shown in Table 2. Gender, presence of mucin, dirty necrosis and age showed similar patterns of immunomarker expression in the two markers. Associations between each immunomarker and the clinicopathological features did not reach statistical significance except those between EpCAM expression and lymphocytic infiltrates in the tumour and between E-cadherin and tumour grade. Seventy-five percent of the tumours with TIL and 67% of cases with Crohn-like lymphocytic aggregates showed EpCAM overexpression (*p* < 0.025; *p* < 0.020, respectively). There was a marginally significant association between E-cadherin positivity and tumour differentiation into low and high grade (*p* = 0.050).

## Discussion

Since its discovery, EpCAM has come to be associated with a plethora of roles, including cell adhesion, modulating other adhesion molecules, intracellular signalling, stemness in circulating tumour cells and as a drug target [11, 18]. Except for its role as an adhesion molecule which has had dual perspectives, most of its understood actions point to it being a poorer prognostic factor in carcinomas. Our data suggest a pattern of expression that supports the latter opinion. We evidently showed that the biomarker was remarkably higher in tumour tissues compared to adjacent non-tumour colon tissue as has been documented by other authors [19].

Patients whose tumours express EpCAM might benefit from anti-EpCAM targeted therapy. Overall, the present study found lower positive EpCAM expression compared to the finding of 94% (97/110) by Spizzo *et al* [15] among the Swiss, and 97.7% (1158/1186) by Went *et al* [20], in a study of German and Iranian populations, respectively. While Spizzo *et al* [15], reported the combination of TMA and whole tissue sections in their study, Went *et al* [20], used only TMA for their study. Besides sample size, the difference in percentage EpCAM overexpression could be due to larger tumour tissue present in whole tissue sections in the study by Spizzo *et al* [15], and the exclusion of staining intensity in the scoring of immunostaining by Went *et al* [20]. Besides these, another possible explanation for this difference in EpCAM expression is the difference in the studied population. Tutlewska *et al* [21], had earlier suggested that EpCAM expression varies among different populations studied. This has significance for the patients seen in our population as it might suggest a subtle different molecular pathway for these tumours that might influence prognosis and patient stratification for therapeutic purposes. A recent multi-ancestry study comparing molecular characteristics of Nigerian CRC and those of European, other African, South and East Asia and American natives has shown remarkable differences involving certain signature genes such as microsatellite instability profile, BRAF and KRAS genes and this might be similar with our finding on EpCAM among our patients [22].

Patient age and gender in this study showed no difference in the proportion of EpCAM expression. This finding is similar to the study by Kim *et al* [23]. Contrary to the study by Kim *et al* [23], tumour location on the left colon and rectum showed a tendency to overexpress EpCAM in this present study. However, both lacked statistical significance. Other studies have also suggested similar findings [24, 25]. This, therefore, suggests an inherent effect of tumour biology on EpCAM expression devoid of influence from age, gender or tumour location as alluded to above [22].

Tumour histology in CRC often reflects the tumour grade (differentiation). Neither tumour histology nor tumour grade was significantly associated with EpCAM expression in the present study contrary to the study by Went *et al* [20], which found a significant association between EpCAM expression and both tumour histology and tumour grade. Other studies have shown an association with only tumour differentiation, while others found an association with tumour differentiation but not with certain histologic subtypes such as mucinous histology and medullary carcinoma [23, 25]. Yet, there are studies that documented a lack of association with tumour grade as was found in the present study but without comment on tumour histology subtypes [24]. We think that the mix of histological subtypes within a study may influence the outcome of data analysis to the extent that the statistical significance of the findings may be difficult to establish. This position will require further studies to verify.

Existing data on the prognostic role of EpCAM is equivocal, some suggesting a dual role as a tumour suppressor and an oncogene [11, 26]. To evaluate this, we noted that most of the cases were histologically low grade, early TNM stage and had fewer numbers of lymphovascular and perineural invasion. Thus, it follows that more cases of EpCAM overexpression would exhibit a similar profile. Also, E-cadherin loss is an independent factor promoting tumour progression [27–29]. The association between E-cadherin and these prognostic factors in this study mirrors that seen with EpCAM except for the pT stage where E-cadherin positive tumours had twice higher late stage tumours suggesting an additional effect of EpCAM in weakening E-cadherin effectiveness in restraining tumour cell invasiveness. By this mechanism, therefore, EpCAM expression may be an early driver of tumourigenesis in addition to potentiating tumour spread.

This study showed an association between TAL and EpCAM overexpression, that was not seen with E-cadherin but studies describing the association between EpCAM expression and TAL are uncommon thereby making comparison difficult whereas the presence of TIL, (either intraepithelial or at the invasive front of the tumour), has been associated with better survival outcomes in CRC patients [30]. It is known that some of the cell surface markers on CRC cells act as tumour-associated antigens (TAA) and that high levels of these antigens in CRC patients induce anergy in TILs in CRC, thus promoting tumour progression [31]. The increased TIL could be the host’s response in an attempt to overcome the anergy. EpCAM, a well-characterised human TAA has been shown to favour immune evasion by CRC tumour cells by adopting Th-2 responses in preference to Th-1 responses [32]. Overexpression of EpCAM, as a TAA, could explain lymphocyte recruitment in CRC as was seen in this study. This result has implications for therapy as cytotoxic T-cells and natural killer cells could be programmed to recognise and destroy biomarker-bearing CRC cells. There is data suggesting that adoptive chimeric antigen-receptor-modified NK-cells transfected with EpCAM gene selectively targets EpCAM-expressing colon cancer cells with a release of interferon (IFN)-γ, perforin and granzyme-B and specific cytotoxicity [33]. Such finding holds promise for therapy in tumours expressing EpCAM [33]. Novel trials are warranted to explore this further in our environment.

A limitation of this study was the drawback of the use of the TMA technique limiting tumour tissue representation on the histospots and loss of some histospots by lifting during tissue processing. This reduces the number of cases included in the final data analysis. Other authors have observed similar effects [16, 20]. A second possible shortcoming of TMA technique may be the effect of tumour heterogeneity. This means that the histospots studied may not reflect the entire tumour expression of the marker. Despite these, it has been shown that EpCAM immunostaining between whole tissue sections and TMA has good concordance [34]. Thus, our finding may reflect true biomarker expression status. Thirdly, the long storage duration of the FFPE tissues could have attenuated the viability of both biomarkers in the tumour cells. Future studies with more recently obtained tissues might yield high marker expression and this is highly recommended.

## Conclusion

We have shown in this study that the majority of CRC in our population preferentially overexpress EpCAM over E-cadherin and this favours local tumour progression by conferring more invasive properties to the tumour cells even among E-cadherin-expressing tumours. EpCAM-bearing tumours are also more likely to attract TALs whose role needs to be ascertained. Further studies involving a larger sample volume are required to test these findings and amass data that could determine interventions to mitigate the present precarious state of the disease in our region.

## Conflicts of interest

The authors declare that they have no conflict of interest to declare with regard to this study.

## Funding

None.

## Institutional review

This study was reviewed and approved by the University of Ibadan/University College Hospital Ibadan Ethics and Review Board with approval number UI/EC/17/0190.

## Author contributions

USE, GOO and OJO conceived the study. USE and MIM prepared the TMA FFPE tissue blocks. All the authors reviewed the H&E and immunohistochemistry slides and interpreted the data. All the authors contributed in writing and editing the manuscript. All the authors gave approval to the final draft for submission.

## Figures and Tables

**Table 1.** Clinicopathological parameters of the study population and tumours.

Variable (*n*)	Frequency (*f*)	Percentage (%)
Gender (63) Male Female	30 33	47.6 52.4
Location (63) Left Right Missing	35 20 8	55.6 31.7 12.7
Histologic type (63) Adenocarcinoma NOS Mucinous carcinoma Signet ring carcinoma	52 7 4	82.6 11.1 6.3
Tumour grade (52) Well differentiated Moderately differentiated Poorly differentiated Others	23 17 11 12	36.5 27 17.5 19
pT Stage pT1 pT2 pT3 pT4	2 25 25 11	3.2 39.7 39.7 17.5
Lymph node metastasis N0 N1–N2 Nx	34 19 10	54.0 30.1 15.9
Distant metastasis (63) M0 M1	55 8	87.3 12.7
Lymphovascular permeation (63) Yes No	15 48	23.8 76.2
Perineural invasion (63) Yes No	6 55	9.5 87.3
Tumour-infiltrating lymphocytes (TILs) (63) Yes No	12 51	19.0 81.0
Crohn-like lymphocytic aggregate (63) Yes No	21 42	33.3 66.7
Dirty necrosis (63) Yes No	18 45	28.6 71.4
pTNM stage I–II III–IV	39 24	61.9 38.1

pT: Pathological tumour staging of the tumour extent in the wall of the colon or rectum; pTNM: Pathological tumour, node and metastasis staging

**Table 2.** Correlation between immunomarkers and clinicopathological parameters.

		EpCAM			E-cadherin		
Variable		Over-expression	Low expression	*p*	Positive	Negative	*p*
Age (years)	≤60 >60	16 14	20 13	0.422	8 4	28 23	0.459
Gender	Male Female	14 16	16 17	0.923	7 5	26 25	0.646
Tumour location	Right Left	8 17	12 18	0.554	1 10	19 25	0.109
Histological type	Adenocarcinoma NOS Others	25 5	27 6	0.874	11 1	41 10	0.446
Tumour grade	Low High	20 5	20 7	0.612	11 0	29 12	0.050*
pT stage	pT1–pT2 pT3–pT4	15 15	12 21	0.422	4 8	23 28	0.459
Lymph node metastasis	Yes No	9 20	11 18	0.461	4 7	16 31	1.000
Distant metastasis	Yes No	2 28	6 27	0.270	1 11	7 44	0.693
LVI	Yes No	8 22	7 26	0.516	3 9	12 39	1.000
PNI	Yes No	4 26	2 31	0.412	0 12	6 45	0.342
CLA	Yes No	15 15	6 27	0.020*	4 8	17 34	1.000
TIL	Yes No	9 21	3 30	0.025*	1 11	11 40	0.433
Dirty necrosis	Yes No	9 21	9 24	0.689	2 10	16 35	0.482
TNM	I–II III–IV	21 9	18 15	0.287	8 4	31 20	1.000

LVI: lymphovascular invasion, PNI: perineural invasion, TIL: tumour-infiltrating lymphocytes, CLA: Crohn-like lymphocytic aggregates

* indicates values that reached statistical significance.

## References

[1] Sung H, Ferlay J, Siegel RL (2021). Global cancer statistics 2020: GLOBOCAN estimates of incidence and mortality worldwide for 36 cancers in 185 countries. CA Cancer J Clin.

[2] Khougali HS, Albashir AA, Daffaalla HN (2019). Demographic and clinicopathological patterns of colorectal cancer at the National Cancer Institute, Sudan. Saudi J Med Med Sci.

[3] Irabor DO (2014). Diet, environmental factors and increasing incidence of colorectal cancer in Nigeria. Ann Niger Med.

[4] Benedix F, Kube R, Meyer F (2010). Comparison of 17,641 patients with right- and left-sided colon cancer: differences in epidemiology, perioperative course histology, and survival. Dis Colon Rectum.

[5] Abdulkareem FB, Abudu EK, Awolola NA (2008). Colorectal carcinoma in Lagos and Sagamu, Southwest Nigeria: a histopathological review. World J Gastroenterol.

[6] Irabor DO, Afuwape OO, Ayandipo OO (2014). The present status of the management of colon and rectal cancer in Nigeria. J Cancer Res.

[7] Craig SEL, Brady-Kalnay SM (2011). Cancer cells cut homophilic cell adhesion molecules and run. Cancer Res.

[8] Herlyn M, Steplewski Z, Herlyn D (1979). Colorectal carcinoma-specific antigen: detection by means of monoclonal antibodies. Proc Natl Acad Sci U S A.

[9] Litvinov SV, Balzar M, Winter MJ (1997). Epithelial cell adhesion molecule (Ep-CAM) modulates cell–cell interactions mediated by classic cadherins. J Cell Biol.

[10] Jeanes A, Gottardi CJ, Yap AS (2008). Cadherins and cancer: how does cadherin dysfunction promote tumor progression?. Oncogene.

[11] Gires O, Pan M, Schinke H (2020). Expression and function of epithelial cell adhesion molecule EpCAM: where are we after 40 years?. Cancer Metastasis Rev.

[12] Nagtegaal I, Arends M, Salto-Tellez M (2019). Colorectal adenocarcinoma. WHO Classification of Tumours of Digestive System.

[13] Brierley JD, Gospodarowicz MK, Wittekind C (2017). Colon and rectum. TNM Classification of Malignant Tumours.

[14] Kononen J, Bubendorf L, Kallionimeni A (1998). Tissue microarrays for high-throughput molecular profiling of tumor specimens. Nat Med.

[15] Spizzo G, Fong D, Wurm M (2011). EpCAM expression in primary tumour tissues and metastases: an immunohistochemical analysis. J Clin Pathol.

[16] Bruun J, Kolberg M, Nesland JM (2014). Prognostic significance of Î^2^-catenin, E-cadherin, and SOX9 in colorectal cancer: results from a large population-representative series. Front Oncol.

[17] Karatzas G, Karayiannakis AJ, Syrigos KN (1999). E-cadherin expression correlates with tumor differentiation in colorectal cancer. Hepatogastroenterology.

[18] Wang M, Sun R, Zhou X (2018). Epithelial cell adhesion molecule overexpression regulates epithelial-mesenchymal transition, stemness and metastasis of nasopharyngeal carcinoma cells via the PTEN / AKT / mTOR pathway. Cell Death Dis.

[19] Yu G, Zhang X, Wang H (2008). CpG island methylation status in the EpCAM promoter region and gene expression. Oncol Rep.

[20] Went P, Vasei M, Bubendorf L (2006). Frequent high-level expression of the immunotherapeutic target Ep-CAM in colon, stomach, prostate and lung cancers. Br J Cancer.

[21] Tutlewska K, Lubinski J, Kurzawski G (2013). Germline deletions in the EPCAM gene as a cause of Lynch syndrome – literature review. Hered Cancer Clin Pract.

[22] Alatise OI, Knapp GC, Sharma A (2021). Molecular and phenotypic profiling of colorectal cancer patients in West Africa reveals biological insights. Nat Commun.

[23] Kim JH, Bae JM, Song YS (2016). Clinicopathologic, molecular, and prognostic implications of the loss of EPCAM expression in colorectal carcinoma. Oncotarget.

[24] Kuhn S, Koch M, Nubel T (2007). A complex of EpCAM, claudin-7, CD44 variant isoforms, and tetraspanins promotes colorectal cancer progression. Mol Cancer Res.

[25] Liu D, Sun J, Zhu J (2014). Expression and clinical significance of colorectal cancer stem cell marker EpCAMhigh/CD44+ in colorectal cancer. Oncol Lett.

[26] van der Gun BTF, Melchers LJ, Ruiters MHJ (2010). EpCAM in carcinogenesis: the good, the bad or the ugly. Carcinogenesis.

[27] Tsanou E, Peschos D, Batistatou A (2008). The E-cadherin adhesion molecule and colorectal cancer. A global literature approach. Anticancer Res.

[28] Stadler M, Scherzer M, Walter S (2018). Exclusion from spheroid formation identifies loss of essential cell-cell adhesion molecules in colon cancer cells. Sci Rep.

[29] Janiszzewska M, Primi MC, Izad T (2020). Cell adhesion in cancer: beyond the migration of single cells. J Biol Chem.

[30] Belov L, Zhou J, Christopherson RI (2011). Cell surface markers in colorectal cancer prognosis. Int J Mol Sci.

[31] Zbar APÃ (2004). The immunology of colorectal cancer. Surg Oncol.

[32] Ziegler A, Heidenreich R, Braumu H (2019). EpCAM, a human tumor-associated antigen promotes Th2 development and tumor immune evasion. Blood.

[33] Zhang Q, Zhang H, Ding J (2018). Combination therapy with EpCAM-CAR-NK-92 cells and regorafenib against human colorectal cancer models. J Immunol Res.

[34] Fong D, Seeber A, Terracciano L (2014). Expression of EpCAM MF and EpCAM MT variants in human carcinomas. J Clin Pathol.

